# V2VSL: Infrastructure-Free, Decentralized Variable Speed Limit Control

**DOI:** 10.1007/s42421-026-00153-9

**Published:** 2026-04-17

**Authors:** Kevin Riehl, Davide Pusino, Anastasios Kouvelas, Michail A. Makridis

**Affiliations:** Traffic Engineering Group, Institute for Transport Planning and Systems, ETH Zürich, Stefano Franscini Platz 5, 8093 Zurich, Switzerland

**Keywords:** Variable speed limit, Decentralized control, Consensus algorithm, Gossip algorithm, Bellmann control

## Abstract

Traffic congestion is a pertinent issue on highways, with severe consequences on environment, economy, and quality of life. Variable speed limit control can help avoid traffic jams before congestion forms, as vehicles upstream are required to decelerate at times to stop emerging congestion from propagating and expanding. This work proposes a fully decentralized, model-free, and infrastructure-free approach to variable speed limit control—V2VSL—that employs connected vehicles as communication infrastructure, as moving sensors, and as actuators. Dedicated short range communication, consensus algorithm and gossip algorithm protocols, and a Bellman controller are components of this approach. At the example of three highway bottleneck scenarios, performance is assessed by traffic micro-simulations, that show the approach is robust to gaps between platoons and capable of recovering from periods of disconnection. The proposed method achieves significant improvements in traffic states, with up to 15% higher speeds, 5% lower density, and 8% higher flows. These traffic improvements become significant at a compliance rate as low as 25%, making the method potentially viable in near-term mixed traffic environments with partial CAV penetration. V2VSL achieves efficiency gains comparable to centralized VSL systems, but without requiring roadside infrastructure, detailed traffic models, or centralized communication. An open-source implementation and computational results are provided as SUMO simulation with Python on GitHub: https://github.com/DerKevinRiehl/decentralized_vsl/.

## Introduction

Traffic congestion on highways is a severe problem, as it causes longer travel times, increased fuel consumption, more emissions, reduced productivity, and increased frustration among drivers (Khulbe et al. 2023; Bagabaldo et al. 2024; Nguyen et al. 2024). According to INRIX Global Traffic Scorecard,^1^ each American driver loses 51 h per year on average due to congestion on highways, which translates to $546 in wasted fuel consumption, and $869 wasted time. While accidents, construction zones, and challenging weather conditions count amongst the most common causes of traffic congestion on highways (Mahmud et al. 2012), traffic jams arise for many reasons. Bottlenecks, such as reduced number of lanes, and on- and off-ramps, can cause congestion (Daganzo 1997). Traffic congestion can also occur at the absence of any bottleneck, due to aggressive, non-cooperative human driving behavior when lane-changing and car-following (phantom traffic jams) (Sugiyama et al. 2008). Overly-aggressive, selfish, dynamic (re-)routing through the use of navigation apps can amplify congestion spread dynamics and over-concentration, leading to more congestion (Bagabaldo et al. 2024).

The slower-is-faster effect (Gershenson and Helbing 2015) suggests vehicles upstream should decelerate (slow down) to enable conflict resolution downstream fast enough to avoid congestion forming. This idea is implemented in variable speed limit control (VSL). VSL dynamically adjusts speed limits on highways to improve traffic flow and prevent congestion (Fang et al. 2023). If implemented correctly, VSL can be an effective countermeasure against congestion on freeways, and significantly improve throughput and safety (Khondaker and Kattan 2015). In practice, VSL can be challenging, due to complexities in real-time measurement of traffic and weather conditions, infrastructural requirements for sensors and electronic speed signs, public acceptance, and driver compliance (Yuan et al. 2022).

The advent of connected vehicles (CVs), automated vehicles (AVs), and both (CAVs) introduces new opportunities to this context and allows the employment of vehicles both as moving actuators—to harmonize traffic flow and alleviate congestion—and as moving sensors (Du et al. 2023). In previous studies, real-time traffic state information (flow, density, speed) is used to inform upstream vehicles that comply with a state feedback controller to reduce their speed if necessary (Du et al. 2023; Tajdari and Roncoli 2023). These previous approaches, however, rely on a communication and sensing infrastructure, accurate traffic state measurements, and model knowledge of the fundamental diagram.

Learning-based approaches in the form of reinforcement learning and multi agent reinforcement learning showcased the potential of decentralized VSL, however real-world implementation remains challenging due to costly onboard computation and communication requirements (Kušić et al. 2020; Fang et al. 2023; Rhanizar and El Akkaoui 2024).

In this work, we address the gap between infrastructure-dependent VSL systems and the emerging opportunities of connected vehicles by proposing V2VSL—a fully decentralized, model-free, and infrastructure-free approach to VSL. The proposed approach requires no infrastructure, and solely builds upon connected vehicles, meaning the ability for vehicle-2-vehicle(V2V) communication. V2VSL leverages *dedicated short range communication* (DSRC) (Kenney 2011) to implement a decentralized communication infrastructure, and makes use of vehicles as moving sensors for decentralized speed estimation with a combination of discrete-time consensus (Zhu and Martínez 2010) and gossip algorithms (Boyd et al. 2005; Dimakis et al. 2010) communication protocols. Finally, compliant vehicles that follow suggestions act according to a Bellman, bang-bang control law (Bellman et al. 1956; Sonneborn and Van Vleck 1964), which does not require an understanding of the system model at any time.

The method is evaluated at the example of a highway bifurcation bottleneck. The results of conducted micro-simulations show, that the controlled approach can achieve significant improvements in traffic (+15% speed, -5% density, and +8% flow) when compared with an uncontrolled situation, even though the decentralized communication does not guarantee recent and accurate speed estimates at all times. The results are consistent for different compliance rates, where at least 25% of vehicles must participate in the control to achieve a significant traffic improvement—making V2VSL potentially viable in near-term mixed traffic environments with partial CV penetration.

This feasibility study contributes to the literature on VSL control and demonstrates, that the proposed V2VSL approach achieves traffic efficiency improvements of a similar order of magnitude to those reported for centralized, infrastructure-based VSL systems in the literature, while relying solely on decentralized, lightweight V2V communication and vehicle-level control. These results do not imply strict performance equivalence under identical conditions, but demonstrate that infrastructure-free, decentralized control can yield comparable congestion-mitigation benefits without the related efforts and costs of centralized, or reinforcement-learning based approaches. The implementation and simulation material can be found on the project’s repository https://github.com/DerKevinRiehl/decentralized_vsl/.

The rest of this work is organized as follows. Section “Literature Review” reviews connected vehicle technologies and technology standards, previous works on VSL, and consensus algorithm based control. Section “Methods” outlines the communication infrastructure, sensing using consensus algorithms, and the Bellman control law of compliant vehicles. Section “Results and Discussion” presents the results of simulations, and discusses their implications. The work concludes with a summary of the main findings and elaborations on future works in Sect. “Conclusions”.

## Literature Review

In this section, we will review related literature on connected vehicle technologies, variable speed limit control, consensus algorithms for control in general and for traffic control in particular, and (reinforcement-)learning based approaches to VSL. This review allows to put the proposed V2VSL into context with previous efforts.

### Connected Vehicle Technologies and Technology Standards

The advent of connected vehicle technologies represents one of the most significant paradigm shifts in traffic management and control over the past two decades. Connected and automated vehicles (CVs, AVs, and CAVs) rely on vehicle-to-vehicle (V2V), vehicle-to-infrastructure (V2I), and broader vehicle-to-everything (V2X) communication to exchange information, such as speed, acceleration, location, and road conditions in real time. This capability enables cooperative applications that can enhance safety, efficiency, and sustainability in transportation systems (Talebpour and Mahmassani 2016; Du et al. 2023).

Two major communication technologies underpin these systems: dedicated short-range communication (DSRC) and cellular vehicle-to-everything (C-V2X). DSRC, standardised under IEEE 802.11p, was initially promoted as the primary technology for V2V and V2I communication, offering low-latency broadcast suitable for safety-critical applications (Kenney 2011). However, despite significant pilot deployments (e.g., the Safety Pilot Model Deployment in the United States), DSRC adoption has remained limited. In contrast, C-V2X, which leverages cellular networks (4 G LTE and increasingly 5 G), has gained momentum in recent years due to its scalability, broader coverage, and strong industry backing (Garcia-Roger et al. 2020; Garcia et al. 2021; Husain et al. 2022).

Standardisation efforts by bodies such as the European Telecommunications Standards Institute (ETSI) and the 3rd Generation Partnership Project (3GPP) are shaping the deployment of V2X technologies globally. ETSI ITS-G5 represents the European counterpart to DSRC, while 3GPP has introduced side-link communication in Release 14 and beyond to support direct V2V communication without relying on cellular base stations (Boban et al. 2018).

These technologies enable a wide range of applications, including cooperative adaptive cruise control, platooning, and eco-driving strategies (Wang et al. 2014, 2016), as well as shared, decentralized traffic state measurement and prediction (Gavric and Stevanovic 2024; Kumar et al. 2024; Nguyen et al. 2024; Jin et al. 2024b; Bhattacharyya et al. 2025).

For speed management, V2V communication can be used to propagate traffic state information upstream, thereby enabling proactive interventions before congestion materialises. Importantly, the reliability, latency, and penetration of communication technologies determine the feasibility of decentralised control approaches such as the proposed V2VSL approach.

### Variable Speed Limit Control

Variable speed limit (VSL) control has long been studied as a strategy to mitigate traffic congestion, improve safety, and reduce environmental impacts. The principle is to dynamically adjust speed limits based on prevailing traffic, weather, or incident conditions, with the aim of harmonising flow and preventing breakdown (Papageorgiou et al. 1983).

Early VSL systems were infrastructure-based, relying on loop detectors, roadside sensors, and gantries with electronic speed displays. For example, VSL has been implemented extensively on European motorways, such as the M25 in the United Kingdom and the Autobahn network in Germany, often as part of managed motorway systems (Schick 2003; Nezamuddin et al. 2011). Evaluations of these systems have shown improvements in throughput and reductions in accident rates, though effectiveness is highly dependent on driver compliance and system design (Khondaker and Kattan 2015; Asadi et al. 2021).

A large body of research has focused on control algorithms for VSL. Rule-based and feedback control approaches dominate early work, where speed limits are set according to measured flow-density states and threshold rules (Smulders 1990; Papageorgiou et al. 1983). More advanced methods include model predictive control (MPC) (Hegyi et al. 2005) and reinforcement learning (Gregurić et al. 2020), which seek to optimise system-wide performance metrics such as travel time, emissions, or safety. A growing branch of research extends section-based to lane-specific speed limits, which is commonly known as differential VSL (Liu and Shi 2018; Jin et al. 2024a).

The rise of CVs introduces new opportunities for VSL. Instead of relying on roadside infrastructure, CVs can disseminate and comply with dynamic speed advisories in a decentralised manner (Tajdari and Roncoli 2023). Research has demonstrated that even a modest penetration of CVs can stabilise traffic flow and suppress the formation of stop-and-go waves, acting as “mobile actuators” (Stern et al. 2018; Du et al. 2023). However, challenges remain in ensuring compliance, integrating human-driven vehicles, and coping with uncertainties in sensing and communication.

### Consensus Algorithms for Control and Traffic Applications

Consensus algorithms are a cornerstone of distributed control theory, allowing multiple agents to agree on a common value (e.g., speed, position, or state estimate) through local interactions (Olfati-Saber et al. 2007). Originally developed in computer science and control engineering, consensus protocols underpin a wide range of networked systems, from sensor fusion to distributed robotics.

In their simplest form, consensus algorithms iteratively update each agent’s state as a weighted average of its neighbours, converging to a global average under mild connectivity conditions (Ren and Beard 2005). Variants, such as gossip algorithms (Boyd et al. 2005; Dimakis et al. 2010) enable consensus through asynchronous, pairwise communication, which is particularly relevant for vehicular networks where connectivity may be intermittent.

Consensus theory has been extended to address robustness, convergence speed, and time delays, all of which are critical for real-world applications. For example, protocols have been designed to tolerate packet loss, switching topologies, or adversarial agents (Nedić et al. 2018). In control applications, consensus is often combined with distributed optimisation or formation control, enabling multi-agent systems to coordinate behaviours without centralised decision-making (Olfati-Saber et al. 2007).

The application of consensus algorithms to transportation has gained attention as a natural framework for decentralised coordination among vehicles. One prominent strand of research is platooning, where consensus is used to align the speeds and headways of vehicles in a convoy/platoon. Early studies demonstrated that consensus-based controllers can maintain string stability and improve fuel efficiency (Elliott et al. 2019; Abdelkader et al. 2021; Ahmed et al. 2022). More recent work integrates vehicle dynamics and communication constraints, showing that V2V consensus can enhance robustness compared to traditional adaptive cruise control (Rajamani 2012).

Beyond platooning, consensus has been applied to traffic signal control (Tan et al. 2020; Zarindast et al. 2024), where distributed agents representing intersections coordinate to optimise network-level performance (Le et al. 2015). In the context of highway traffic management, consensus algorithms have been explored for cooperative merging, distributed ramp metering, and speed harmonisation (Seliman et al. 2020; Kumaravel et al. 2021). For example, Rios-Torres and Malikopoulos (2016) proposed a consensus-based approach for cooperative merging that significantly reduced disturbances at on-ramps.

The integration of consensus algorithms with VSL control is less well explored, but promising. By enabling vehicles to exchange and average speed estimates, consensus can provide decentralised, infrastructure-free sensing of traffic states. This aligns with findings that partial vehicle penetration can already yield measurable benefits if consensus is fast and robust enough (Zhao et al. 2023). The key challenges include ensuring stability in heterogeneous traffic, coping with communication delays, and integrating human-driven vehicles with CAVs.

### (Reinforcement-)Learning Based Approaches to VSL

Reinforcement learning (RL) has emerged as a prominent paradigm for VSL, offering adaptability to diverse motorway conditions without requiring explicit traffic models (Kušić et al. 2020). While RL simplifies control compared to computationally intensive optimal methods (Schmidt-Dumont and Vuuren 2015), it faces challenges in real-world deployment, including high training costs and sim-to-real gaps (Zhang et al. 2024, 2025). Besides, RL facilitates combined problem approaches, such as the combination of ramp metering and VSL (Schmidt-Dumont and Vuuren 2015).

A growing trend towards multi-agent RL (MARL) enables decentralised coordination, treating either vehicles or road sections as agents (Fang et al. 2023; Zhang et al. 2023, 2024), with reviews tracing its evolution from single- to multi-agent strategies (Rhanizar and El Akkaoui 2024). MARL eliminates central coordinators and infrastructure needs, yet demands high compliance, cooperation, and onboard computation—contrasting sharply with our lightweight, analytic V2V gossip consensus and bang-bang control for mixed fleets. Furthermore, not only communication on shared road state perception, but also parameter sharing when learning collectively, makes those approaches not only computationally, but also communicatively expensive.

The consensus-based control architecture proposed in this paper provides a deterministic, computationally, and communicatively efficient alternative. The approach leverages connected vehicles to share minimal local information and compute decentralised control actions based on consensus rules, resulting in a system that is both robust and scalable. The bang-bang control policy, derived from Bellman-optimality principles, ensures rapid convergence and stability without requiring extensive training or high computational resources. Consequently, while RL-based methods emphasize learning adaptivity through exploration to maximize performance, the proposed approach focuses on real-time implementability and reliability under realistic communication and infrastructure constraints minimizing required efforts and costs—ideal for near-term, mixed fleets.

## Methods

This section presents the proposed method V2VSL and its components—V2V communication protocol, speed estimation and consensus algorithm, VSL control law, and implementation considerations.

### Connected Vehicles and Freeway

**Fig. 1:**
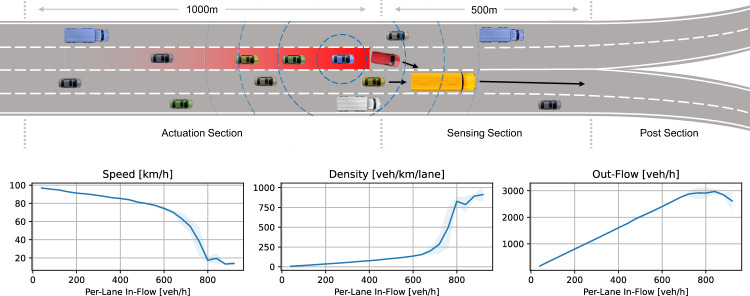
Congestion formation at highway bifurcation. Impaired speed, density, and out-flow of ROI section (sensing) for increasing per-lane in-flows

We consider a population of *n* vehicles on a multi-lane freeway segment. The freeway segment consists of three sections: actuation section, sensing section, and post section, as exemplified in Fig. 1. Vehicles in the actuation section serve as mobile actuators, follow a control law, and actively reduce their speeds to enable decongestion in the bottleneck. Vehicles in the sensing section serve as mobile sensors and estimate the average vehicle speed on this section $v^\textrm{ROI}$. This section is also called the region of interest (ROI), it is where the congestion due to the bottleneck (turbulence due to lane changing manoeuvrers at the bifurcation) happens. While vehicles in the previous two sections communicate, vehicles in the post section do not participate in any communication, sensing, or actuation. Each vehicle *i* has a physical maximum speed ${\tilde{v}}_i^{\max }$, a real speed $v_i$ and a section $s_i$ that represents the section it is located on (assuming they are equipped with modern navigation and localization systems enabling them access to their own current position).

### Communication Infrastructure

**Fig. 2:**
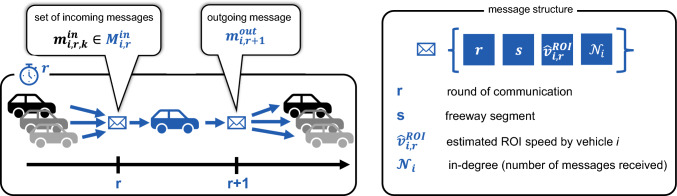
V2VSL Communication Protocol. The V2VSL communication protocol application layer is based on the physical DSRC communication layer, and is conducted as a round based communication. Each round, messages from neighbouring vehicles *k* (within the communication distance) are received, processed, and then a message is send out to all neighbouring vehicles. Each message consists of four fields, including timestamp (round), position (segment), estimate (of average vehicle speed in sensing section), and the in-degree (number of messages received in this round)

Our method employs connected vehicles that communicate vehicle-2-vehicle (V2V), leveraging DSRC technology. The V2VSL communication protocol is summarized in Fig. 2. We assume a share γ of all vehicles is connected, and a maximum possible communication distance $d_c = 200$ m. The communication takes place in rounds, and each round takes $t_r$ time. During each round *r*, each vehicle sends out a message $m_{i,r}^\textrm{out}$ (in case it has a speed estimate available), and receives a set of messages $m_{i,r,k}^\textrm{in} \in {\mathcal {M}}_{i,r}^\textrm{in}$ from surrounding vehicles *k*. At the end of each round, the received messages are processed, and new messages to send out are prepared. Each vehicle’s message consists of four fields: (A) “timestamp”, (B) “section”, (C) “value”, and (D) “in-degree”. Field (A) is populated with the current timestamp, field (B) is populated with the vehicle’s current section, field (C) is populated with the vehicle’s current estimate ${\hat{v}}_{i,r}^\textrm{ROI}$, and field (D) is populated with the vehicle’s in-degree ${\mathcal {N}}_i$, which represents the number of received messages. These fields are used by the gossip and consensus algorithms during speed estimation.

### Speed Estimation and Communication Protocol

**Fig. 3:**
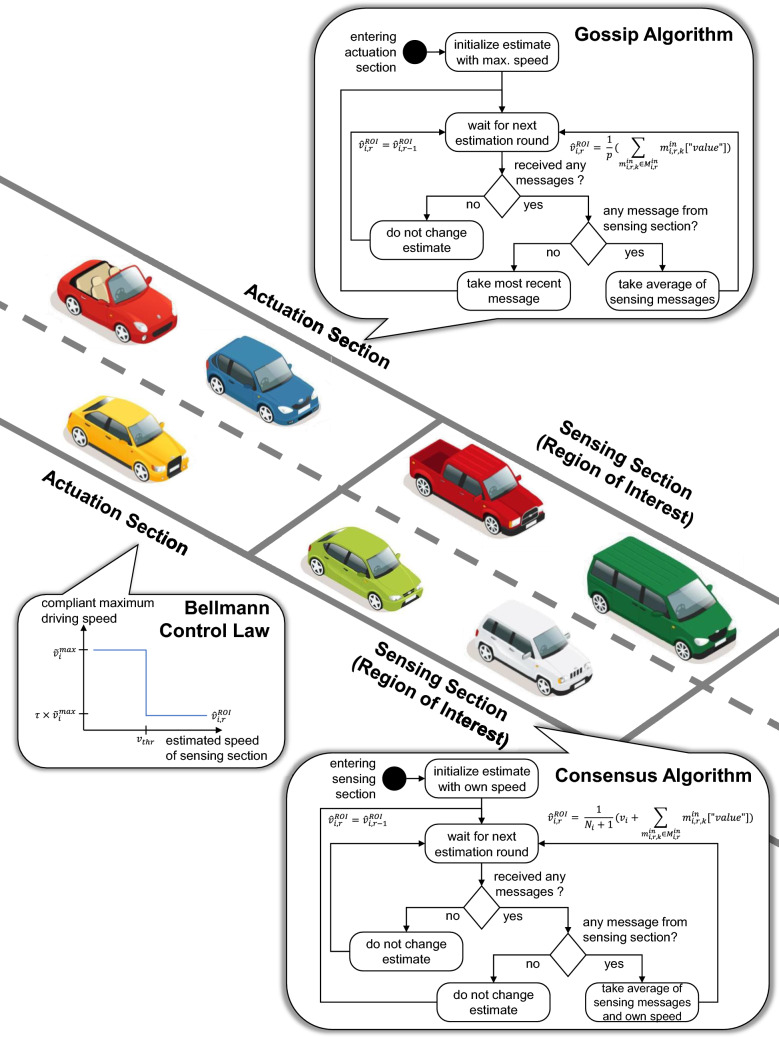
Speed estimation algorithm and VSL control law. Vehicles in the sensing section (region of interest) follow the consensus algorithm to estimate the average vehicle speed. Vehicles upstream in the actuation section follow the gossip algorithm to estimate the average vehicle speed, and regulate their desired driving speed using a Bellman control law

Vehicles in the sensing section estimate the speed state of the ROI $v^\textrm{ROI}$ using a discrete-time average consensus algorithm, as shown in Fig. 3. We assume, for tractability, that traffic across lanes can be represented by a single average speed as the highway segment is before a bifurcation where several lane changing manoeuvrers happen—though in reality outer lanes may carry faster vehicles than inner lanes. Vehicles in the sensing section determine their estimate of the ROI speed ${\hat{v}}_{i,r}^\textrm{ROI}$ as the weighted average of their own speed and the received estimates from surrounding vehicles in the sensing section as follows:

1 $$\begin{aligned}&{\hat{v}}_{i,r}^\textrm{ROI} = \frac{1}{{\mathcal {N}}_i+1} ( v_i + \sum _{m_{i,r,k}^\textrm{in} \in {\mathcal {M}}_{i,r}^\textrm{in}}m_{i,r,k}^\textrm{in} \left[ \mathrm{"value" }\right] ) \end{aligned}$$

Vehicles in the actuation section communicate their received estimates via a gossip algorithm with each other, and therefore back-propagate information on received estimates from the sensing section, as shown in Fig. 3. They determine their speed estimates as described in the three following cases.

(i) In case the vehicle did not receive any message from surrounding vehicles in the communication round, it will stick to its previous estimate:
  2 $$\begin{aligned}&{\hat{v}}_{i,r}^\textrm{ROI} = {\hat{v}}_{i,r-1}^\textrm{ROI}. \end{aligned}$$
(ii) In case the vehicle received at least one message from surrounding vehicles of the sensing section, it will consider only those *p* messages from the sensing section and determine its speed estimate as the average of the received speed estimates:
  3 $$\begin{aligned}&{\hat{v}}_{i,r}^\textrm{ROI} = \frac{1}{p} \left( \sum _{m_{i,r,k}^\textrm{in} \in {\mathcal {M}}_{i,r}^\textrm{in}} m_{i,r,k}^\textrm{in} \left[ \mathrm{"value"} \right] \right) . \end{aligned}$$
(iii) In case the vehicle received messages from surrounding vehicles of the actuation section only, it will determine its estimate as the most recent available estimate from all received messages.

If the resulting estimate from any of the cases above exceeds a certain maximum considered estimation age $a_{\max }$, the vehicle forgets previous estimates and does not possess an estimate. Vehicles that just entered the sensing section will reset their speed estimate to their current speed.

Vehicles in the sensing section always have a speed estimate of the ROI (at least determined by their own speed), while vehicles in the actuation section do not always have a speed estimate.

### Control Law

Vehicles in the actuation section—that are connected and compliant—reduce their desired driving speed according to the recommendation of the VSL control law, as shown in Fig. 3. We assume a share γ of all vehicles to be connected and compliant (this can be both CAVs, human-driven CVs, or human drivers that simply activate a specific advanced driver assistance system in addition to adaptive cruise control). Each connected and compliant vehicle *i* applies a simple Bellman, two-point, bang-bang control law (Bellman et al. 1956; Sonneborn and Van Vleck 1964): it either keeps its maximum speed $v_i^{\max }$ or reduces it slightly when estimated speeds in the region of interest ${\hat{v}}_i^\textrm{ROI}$ fall below a threshold:

4 $$\begin{aligned}&v_i^{\max } = \left\{ \begin{matrix} {{\tilde{v}}}_i^{\max } & \text {\;\; if \;\;} {\hat{v}}_i^\textrm{ROI} \ge v_\textrm{thr} \\ \tau \times {{\tilde{v}}}_i^{\max } & \text {\;\; if \;\;} {\hat{v}}_i^\textrm{ROI} < v_\textrm{thr} \end{matrix}\right. \end{aligned}$$

If the speed estimate ${\hat{v}}_i^\textrm{ROI}$ drops below a certain threshold speed $v_\textrm{thr}$, vehicles will consider a reduced maximum speed, which is defined by the speed reduction factor $\tau \in \left[ 0, 1 \right]$.

### Highway Division and Implementation Considerations

#### Highway as Sequence of Problem-Zones

VSL is particularly effective upstream of problem zones—localized roadway features that induce traffic disturbances, turbulence, and capacity drops. Such problem zones include on-ramps and merging areas, lane drops, and off-ramps or highway bifurcations where intensive lane-changing occurs. A freeway corridor may contain multiple such problem zones, each acting as a potential congestion trigger.

In the proposed V2VSL framework, each problem zone is treated independently and decomposed into three contiguous control segments: a sensing section, an actuation section, and a post section. This decomposition is not arbitrary; it reflects the distinct functional roles required for decentralized state estimation, upstream intervention, and downstream evaluation under V2V communication constraints.

#### Sectional Composition of Problem-Zones

*Sensing section (region of interest, ROI):* The sensing section is co-located with the problem zone itself and corresponds to the region where congestion first manifests. Vehicles within this region participate in a discrete-time average consensus algorithm to estimate the local mean traffic speed. By focusing sensing on the immediate vicinity of the disturbance source, the estimated state remains representative of the emerging bottleneck dynamics, while avoiding contamination from upstream traffic conditions that are less relevant to imminent congestion formation.

*Actuation section:* The actuation section is located upstream of the sensing section and contains vehicles that apply the VSL control law. Vehicles in this region act as mobile actuators, reducing their desired speeds when congestion is detected downstream. This upstream placement allows speed harmonization to propagate gradually through car-following interactions, mitigating shock wave formation before vehicles reach the problem zone. Information from the sensing section is relayed upstream via gossip-based communication, enabling decentralized control without infrastructure support.

*Post section:* Vehicles in the post section do not participate in sensing, communication, or actuation. This section provides spatial separation between consecutive problem zones along a corridor and allows downstream traffic conditions to be observed without feedback interference. From an operational perspective, it also reflects that speed interventions are unnecessary once vehicles have passed the bottleneck.

#### Sensitivity and Generalizability

The performance of V2VSL depends on the interaction between section lengths, traffic conditions, and communication parameters. Rather than being tied to a specific geometry, the design follows general principles: the sensing section must allow sufficient information exchange for consensus under expected densities; the actuation section must provide enough upstream distance for control effects to materialize without excessive information latency; and the post section must ensure separation between control zones. These principles apply across different types of problem zones, including on-ramps, lane drops, and bifurcations, and can be satisfied by scaling section lengths according to typical speeds, densities, and V2V communication capabilities.

#### Retrieval of Parameters for Problem-Zones

In practice, problem zones and their associated V2VSL configurations could be encoded as geo-fenced regions in digital map services, similar to how speed limits or road attributes are currently distributed. Vehicles approaching a known problem zone would retrieve the corresponding configuration and automatically adapt their behaviour, enabling network-wide deployment of decentralized VSL without centralized sensing or control infrastructure.

## Results and Discussion

This feasibility study leverages traffic microsimulations to assess the performance and characteristics of the proposed V2VSL methodology. In this section, we (i) present three different micro-simulation scenarios that were used for evaluation and benchmark, (ii) discuss dedicated experiments that were conducted for the design and evaluation of the communication system and controller, (iii) analyse control performance, (iv) discuss convergence guarantees of the consensus algorithm, and (iv) conduct an extensive benchmark with alternative control designs.

### Simulation Design

**Fig. 4:**
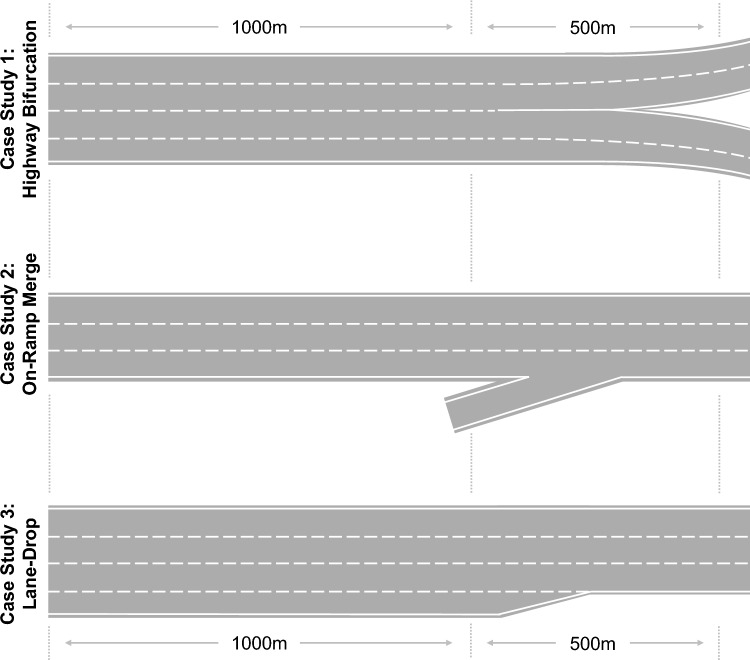
Three highway bottleneck case studies. These three case studies are used for the feasibility assessment and evaluation of the V2VSL methodology. Each of the case studies represents a common reason for congestion on highways

Congestion on highways can arise for a multitude of reasons, where bottlenecks are one of the major reasons that can be addressed with control techniques, such as VSL. In this feasibility study, we analyse and demonstrate the performance of V2VSL at the example of three case studies: bifurcation, on-ramp merge, and lane-drop, as shown in Fig. 4. These three case studies are outlined in the following, accompanied by computational implementation details of the traffic microsimulation.

#### Highway with Bifurcation-Turbulence Bottleneck

This case study exemplifies the congestion formation at a highway bifurcation with four lanes. As vehicles need to perform their lane changes before the bifurcation, conflicts can occur, slowing down vehicles upstream and causing turbulences and increasing congestion. The red vehicle in Fig. 1 for example needs to change lanes, but as other vehicles on that lane, such as the yellow bus, prevent it from lane changing, it must decelerate, which causes congestion on the vehicles upstream in its lane. For higher levels of in-flowing traffic, the average speed and out-flow in the ROI (sensing section) drops, while the density grows. This case study was used primarily for communication design, control design, the discussion of control performance, and the assessment of convergence guarantees.

#### Highway with On-Ramp Bottleneck

This case study exemplifies the congestion formation at a highway on-ramp. When vehicles enter the highway from the on-ramp, they interfere with the trajectories of cars that are on the highway already, and cause those to slow down or change lanes. Furthermore, the flow of vehicles before the ramp is smaller than after the ramp while the space (number of lanes) stays the same. For these two reasons, congestion can be observed around the merging area of the on-ramp.

#### Highway with Lane and Capacity Drop Bottleneck

This case study exemplified the congestion formation at a highway lane-drop. At the beginning, the number of lanes is four, and is the reduced to three. Due to this reduction, vehicles need to conduct lane-change manoeuvrers to accommodate for the reduced space.

#### Computational Details on Traffic Simulation

We conduct time-discrete micro-simulations of the road network using SUMO (Lopez et al. 2018). Traffic demand is generated stochastically using a Bernoulli process and random lane origin–destination assignment using a uniform distribution. The fleet composition on the highway is assumed to be mixed traffic consisting of the following four vehicle types: cars (55%, max. speed 200 km/h), delivery vehicles (22%, max. speed 200 km/h), omnibuses (11%, max. speed 85 km/h), and trucks (12%, max. speed 130 km/h). We employ a Krauss car-following model (Krauß 1998; Krauß et al. 1997), and an Erdmann lane-changing model (Erdmann 2015), while noting that lane-changing behaviour (e.g., slow trucks in fast lanes) may bias local estimates.

The maximum speed on the highway is assumed to be 100 km/h (which can be violated/exceeded by aggressive driving behaviour up to 25 km/h). Traffic simulation experiments are run at a step-length of 1 s, for 6000 s of simulation time, with an additional 1000 s warm-up time, and repeated 20 times with different random seeds to determine average and standard deviation for traffic fundamentals (speed, density, flow), communication, and control-related statistics.

### Communication Design

**Fig. 5:**
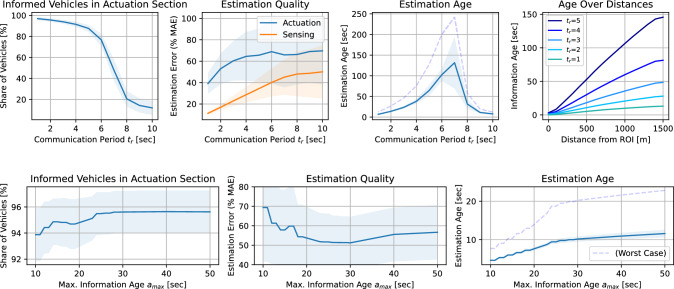
DSRC communication design. Communication period duration $t_r$, maximum estimation age $a_{\max }$, and their effect on ROI speed estimation quality. Plots generated for per-lane in-flows of 720 veh/h

Figure 5 describes the decentralized communication system design. The first row shows the effects of varying the communication period $t_r$ on the ROI speed estimation quality of vehicles in the actuation and sensing section. The mean absolute estimation error (MAE) is used to quantify the quality of vehicles’ estimates.

The estimation quality of vehicles in the sensing section is consistently higher, as these vehicles actively participate in the average consensus algorithm when compared with the vehicles in the actuation section. Vehicles in the actuation section receive information via the gossip algorithm, and the most accurate and recent estimates back-propagate over time from vehicle to vehicle upstream. This results in outdated, delayed (aged) estimate information (latency) the further actuated vehicles are away from the ROI upstream.

For shorter periods $t_r$ (more frequent communication), we observe consistent communication improvements. The share of vehicles in the actuation section that have an estimate available can grow to almost 100%, the age of estimates decreases for all distances of vehicles in the actuation section, and the estimation quality for both actuated and sensing vehicles can be significantly improved.

Smaller maximum estimation ages $a_{\max }$ that are considered by vehicles in the actuation section, can help to reduce the estimates’ age at the cost of the share of vehicles that still have an estimate available and the estimation quality.

Therefore, we finally consider the following parameter combination for the communication infrastructure: every $t_r=2$ s speed estimation, and $a_{\max }=30$s maximum information age. This parameter combination achieves sufficiently high estimation quality, and broad information distribution, while not costing too much DSRC bandwidth, and not demanding too frequent communication from vehicles.

### Control Design

**Fig. 6:**
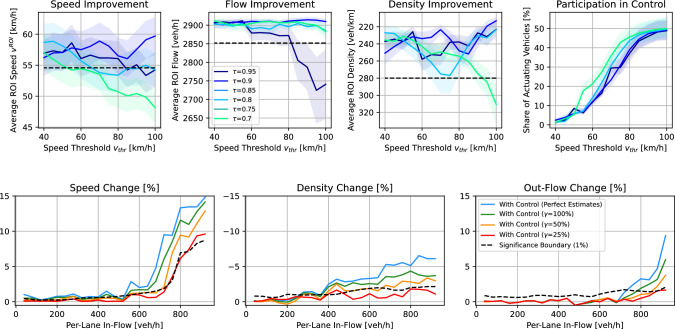
Two-point controller design. Speed control threshold $v_\textrm{thr}$, speed control factor τ, compliance ratio γ, and their effect on ROI traffic improvement. Plots in the first row show traffic improvement for per-lane in-flows of 720 veh/h

Figure 6 (top row) describes the decentralized two-point controller design. The speed threshold $v_\textrm{thr}$ and speed reduction factor τ are varied, with effects on the participation of vehicles in the actuation section, and with mixed findings on improvements in speed, flow, and density. The higher $v_\textrm{thr}$, the more vehicles participate in reducing their speed. Depending on τ improvements in speed, flow, and density can be achieved. The experiments suggest an optimal configuration of $v_\textrm{thr}=80$ km/h and τ=0.9, as this parameter combination achieves the largest and most consistent improvements across all three traffic fundamentals.

Finally, the control performance is evaluated for different per-lane in-flows, and different compliance rates γ. The results can be found in Fig. 6 (bottom row). Significant improvements can be achieved beginning from an in-flow beginning from 720 veh/h. The ROI speed can be increased by 15%, the density can be reduced by 5%, and the out-flow can be increased by 8%. The improvements resemble those of prior works using VSL in a similar context (Du et al. 2023; Tajdari and Roncoli 2023).

Even when estimation temporarily fails (outdated or inaccurate estimates) due to platoon gaps, traffic conditions do not deteriorate severely and the system recovers quickly—demonstrating a form of graceful degradation and supporting that the proposed method achieves comparably similar performance improvements to vehicles with perfect estimates (blue line). The more vehicles comply with the control law (larger γ), the better the control performance. Based on our simulations, at least γ=25% compliance rate is necessary to achieve significant improvements. This is consistent with prior findings employing a centralized sensing and communication approach (Du et al. 2023; Tajdari and Roncoli 2023).

### Control Performance and Impediments

**Fig. 7:**
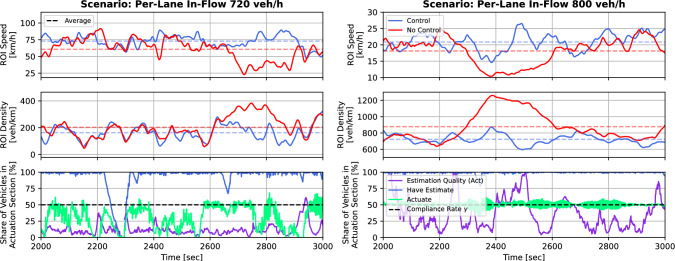
Controller effect on two congestion scenarios. Speed, density, and estimation metrics are shown for high (720 veh/h) and very high (800 veh/h) inflows. The controller ($\gamma =50\%$) can significantly reduce congestion in both cases

Figure 7 presents the control in action for two different in-flow scenarios. When speed drops and density rises without control, one can especially well observe the stabilization introduced by the decentralized, Bellman controller, with its implications on the traffic state.

The participation in actuation and estimation quality depends on the scenario. The estimation quality (measured as relative mean absolute error of the ROI speed estimates) is essentially higher before capacity (left scenario) when compared with at capacity (right scenario). Moreover, before capacity, the control causes actuation of vehicles when it is necessary to decongest, while at capacity an almost permanent actuation of compliant vehicles can be observed. Thus, even a suboptimal communication, with communication failures at sometimes, is just good-enough to achieve significant improvements as it causes action when required.

**Fig. 8:**
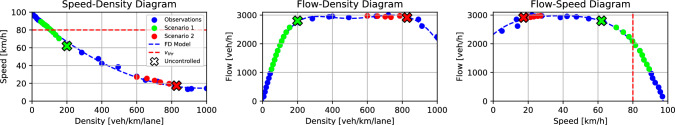
Fundamental diagram of highway bifurcation and control. The fundamental diagram of roads puts the traffic state fundamentals speed, density, and flow into relation with each other. Based on observations, typically, a negative decay between speed and density, and a u-shaped relationship between flow and density, and flow and speed can be observed. The FD models are estimated with a polynomial fit throughout the observations

The left scenario showcases another challenge of the decentralized approach between 2000 s and 2400 s. In this interval, the share of vehicles upstream that have an estimate drops significantly, and estimation quality worsens substantially. Vehicles tend to cluster in platoons. If a gap between two groups of vehicles is larger than the maximum communication distance $d_c$, it acts like an information barrier preventing the flow of information upstream. This causes the communication system, consensus, and gossip algorithm to fail due to the lack of connectivity. Even though the communication and estimation fail during that interval, and therefore no actuation takes place, there are no significant impairments of the traffic state during those times of low density as actuation is not necessary, and the communication system recovers just before control becomes necessary again.

In addition to that, the two scenarios of Fig. 7 demonstrate the correlation of estimation quality, actuation, and the ROI traffic state. In times of a dropping speed and rising density in the ROI, actuation starts to increase after some delay and stays on a high activity level until the traffic state recovers. When estimates worsen, the effects of control on traffic state improvements are disturbed. What’s more, the results of Fig. 7 highlight, that this approach not only improves the speed on average but also homogenizes the traffic over time.

Most existing studies compare traffic control approaches primarily in terms of achieved performance gains. However, such comparisons often neglect the substantial differences in implementation effort, including requirements for infrastructure, sensing fidelity, communication bandwidth, model knowledge, and computational complexity. The results presented here demonstrate that even a highly simplified, bang-bang-style control law combined with decentralized estimation can yield meaningful congestion mitigation effects, suggesting that a favourable performance–effort trade-off can be achieved without complex optimization or learning.

The scenario with perfect state estimation can be interpreted as an upper-bound reference corresponding to an idealized centralized VSL system with complete and noise-free information. The fact that the proposed decentralized V2VSL approach achieves improvements of similar order of magnitude under imperfect and intermittent communication highlights the potential of infrastructure-free control.

### Convergence Guarantees and Influence on Control

Generally, there is no convergence guarantee for the time-discrete average consensus algorithm, as there is an underlying, time-varying communication network topology (switching topology). The vehicles in the sensing section permanently change, due to vehicles entering from the actuation section, and due to vehicles leaving into the post section. Moreover, the underlying average changes over time as well (as the vehicles change their speed continuously).

However, for small communication periods $t_r$ (very frequent communication, $t_r<0.5$ s) the topology can be assumed to be constant over time, and the underlying speed average to be constant for short time periods. Similarly, the estimation quality in the actuation section will improve as well, and the estimation age will shrink to negligible delays with more frequent communication. Therefore, convergence can be guaranteed if and only if the vehicle-2-vehicle communication network graph is strongly connected, as each vehicle includes its own estimate (presence of self-loops). The connectivity of the graph will depend on the density of vehicles in the sensing section and the maximum communication distance $d_c$.

The width of the four-lane highway (16 m) is negligible when compared to its section length (500 m and 1000 m), and therefore a single-lane highway can be assumed in the following discussion. We consider that the arrival of vehicles in the ROI is equivalent to sampling every second, an independent Bernoulli random variable of probability *p*. Assuming a constant speed of *v* for all vehicles, the time delay between two consecutive vehicles entering the ROI can be described as a geometrically distributed, random variable *T*, where *p* equals the vehicle flow per second:

5 $$\begin{aligned}&P(T = t) = (1-p)^{t-1}p. \end{aligned}$$

The distance between two consecutive vehicles can therefore also be described as a geometrically distributed random variable *D*:

6 $$\begin{aligned}&P(D = d) = (1-p)^{\frac{d}{v}-1}p. \end{aligned}$$

Given the previously mentioned assumptions, the convergence of the consensus algorithm can be guaranteed if the communication graph between the vehicles is strongly connected, which is the case if and only if every distance $D_i$ between two consecutive vehicles is smaller or equal than the maximum communication distance $d_c$. The number of vehicles inside the ROI *N* can be determined as the product of density ν and length of the sensing section $l_s$. The convergence guarantee is as follows:

7 $$\begin{aligned} P\left( \text {Strongly Connected}\right)&= P\left( \bigcap _{i=1}^{N-1} D_i \le d_c \right) = \left( P(D_i \le d_c) \right) ^{N-1}\nonumber \\&= \left( 1-\left( 1-p\right) ^{\frac{d_c}{v}-1}\right) ^{N-1}. \end{aligned}$$

**Fig. 9:**
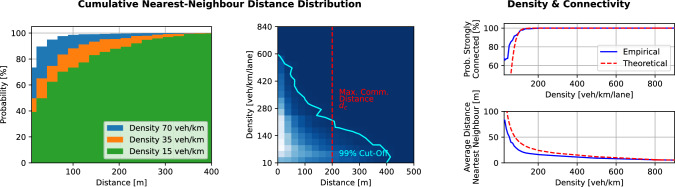
Distance and connectivity. The convergence of the consensus algorithm depends on the strong connectedness of the communication graph. The graph’s connectivity depends on the density of vehicles and the maximum communication distance. Convergence and strong connectedness can only be guaranteed if the distance of vehicles to their nearest neighbours is smaller than the maximum communication distance. The theoretic convergence guarantees match the empiric observations for sufficiently large density

Flow *p* and speed *v* can be determined by the relationships of the fundamental diagram (Fig. 8) for a given density ν. The dependence of the graph’s strong connectedness on density is shown in Fig. 9. The cumulative distance distribution in Fig. 9 (left) empirically supports the assumption of the single lane (empiric distribution with four lanes from simulations follows the theoretical, geometric distribution with one lane).

Depending on the density of vehicles, different distance distributions exist, as shown in Fig. 9 (middle). The distance distributions will determine the probability that all vehicles that lie on the sensing section will form a strongly connected, vehicle-2-vehicle communication topology, as shown in Fig. 9 (right). The geometric distribution of distances is also reflected in the connectivity of the graph depending on the vehicle density. Beginning from a density of as low as 200 veh/km more than 99% of all vehicles have a communication distance to their neighbours that is lower than $d_c$, and therefore span a strongly connected graph. This critical density of 200 veh/km is easily reached on highways in practice, long before traffic congestion is an issue (per lane in-flow of around 720 veh/h), see Fig. 1. It is especially at this critical density that the controller is observed to cause significant improvements to the traffic state, see Fig. 7.

In terms of control, a minimum compliance rate $\gamma =25\%$ suffices, as there are four lanes. For this compliance rate, on average, each lane will have at least one vehicle that slows down, forcing the vehicles behind it to slow down as well. This is, why for this minimum γ we can observe significant improvements in the traffic situation in Fig. 6. Of course, aggressive drivers in a hurry could try to avoid these vehicles by lane-changing manoeuvrers, which gets increasingly harder for larger γ.

A discussion for speed threshold $v_\textrm{thr}$ and speed reduction factor τ is more challenging though, and must be selected for a specific road context. The Bellman controller with $v_\textrm{thr}$ and τ will cause an oscillating between two operating points around the mean speed with control, which can be observed in Fig. 7 and Fig. 8. The amplitudes and frequency of oscillations depend on the traffic state (position on fundamental diagram, FD), τ, $v_\textrm{thr}$, and γ. The share of vehicles that actuate depends on $v_\textrm{thr}$ and the current speed/density, as shown in Fig. 7 (left) and Fig. 8. Once the speed drops below $v_\textrm{thr}$, an increase of the actuating vehicles up to the compliance rate γ can be observed (with some delays).

In the other scenario of Fig. 7 (right), when speed is permanently below $v_\textrm{thr}$, the share of actuating vehicles is permanently around the compliance rate γ. Fig. 8 demonstrates the role of τ; in both scenarios, the speed oscillates with an amplitude of around τ due to the controller. Due to the higher decay in scenario 1 (720 veh/h), the oscillations are larger when compared with scenario 2 (800 veh/h). In this case, tuning $v_\textrm{thr}$ and τ is comparable to varying the controller’s aggressiveness (low $v_\textrm{thr}$ and τ) that acts with a strong control input as soon as a slowdown is estimated, or a more robust controller (high $v_\textrm{thr}$ and τ) that will only act when needed (Fig. 6).

To summarize, the consensus is guaranteed to achieve perfect estimates (assuming highly frequent communication) beginning from a critical vehicle density of around 200 veh/km. The two-point controller achieves significant improvements and transitions the traffic state to slightly oscillating, improving traffic conditions along the fundamental diagram.

### Section Design

In this section, we study the influence of section length design on communication and control performance. The aforementioned communication and control design to determine suitable parameters for V2VSL were conducted assuming a 500 m length of the sensing section, and a 1000 m length of the actuation section, as outlined in Table 1. Here, we try to understand the role of section lengths.

**Table 1:** Parameter design settings for V2VSL

Symbol	Description	Value	Unit
Communication parameter			
$t_r$	Communication period	≤2	s
γ	Share of connected and compliant vehicles	≥25	%
$d_c$	Maximum DSRC communication distance	200	m
$a_{\max }$	Maximum considered information age	30	s
Control parameter			
$v_\textrm{thr}$	Threshold speed (Bellman law)	80	km/h
τ	Speed reduction factor (Bellman law)	90	%
$v_{i,\max }$	Maximum vehicle speed	100	km/h
Section parameter			
$L_\textrm{act}$	Length of actuation section	1000	m
$L_\textrm{sen}$	Length of sensing section	500	m

**Fig. 10:**
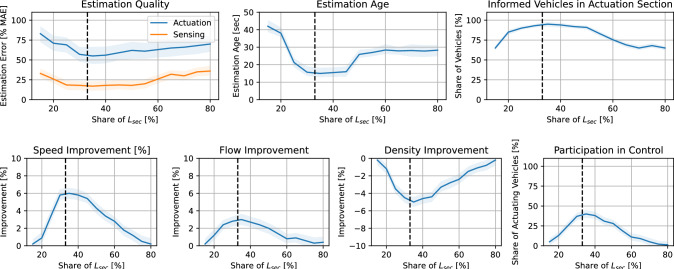
Section design. Impact of section length variation of communication quality and control performance. Plots rendered for traffic of 720 veh/h and τ=75%

We vary the length of sensing $L_\textrm{sec}$ and actuation section $L_\textrm{act}$ and explore implications and communication and control performance. Given the 1.5 km long segment before the bifurcation, we vary the length of the sensing section between 15 and 80%, while the remaining space is dedicated to the actuation section. The results of this analysis are displayed in Fig. 10.

For short sensing section shares (15–20% of the total area), the estimation error is high, indicating insufficient spatial information for accurate state reconstruction. As $L_\textrm{sec}$ increases, the MAE decreases rapidly and reaches its minimum around 30–40%. This suggests that the controller benefits from improved observability when a sufficiently large upstream region is monitored. However, beyond approximately 50%, the estimation error gradually increases again. This degradation can be attributed to the fact that information collected too far upstream becomes less representative of the traffic conditions in the actuation region, thereby reducing effective estimation accuracy. Overall, an intermediate sensing share yields the best estimation performance.

The estimation age follows a trend similar to the estimation error. For short sensing sections, the estimation age is high (exceeding 40 s), indicating infrequent or delayed state updates due to limited vehicle coverage. Increasing the sensing length significantly reduces the estimation age, reaching a minimum around 30–35%, where information updates are both frequent and spatially relevant. When the sensing section becomes too long, the estimation age increases again. Although more vehicles are observed upstream, the information requires additional time to propagate to the actuation region, effectively increasing the age of the utilized estimates.

The share of informed vehicles within the actuation section increases sharply as the sensing region expands from 15% to approximately 35–40%. At this point, nearly all vehicles entering the actuation zone are informed, indicating efficient information dissemination. For larger sensing shares, however, the proportion of informed vehicles gradually declines. This behaviour suggests that excessively long sensing regions shift emphasis upstream and reduce the effective overlap between sensing and actuation.

The macroscopic performance indicators confirm the estimation trends. Speed improvement is negligible for short sensing sections due to poor state awareness and limited control effectiveness. As the sensing share increases, speed gains rise sharply and reach a maximum of approximately 6% around 30–35%. Beyond this region, speed improvements gradually diminish. This indicates that traffic efficiency benefits most when sensing and actuation are balanced, while both under-observation and excessive upstream sensing reduce the achievable performance gains. A similar pattern is observed for traffic flow. Flow improvements increase with sensing length up to approximately 30–40%, where the maximum benefit is obtained. For larger sensing shares, the gains decrease steadily. This suggests that the controller’s ability to stabilize and homogenize traffic flow depends critically on receiving sufficiently localized and timely information. When sensing extends too far upstream, the reduced effectiveness of actuation diminishes the achievable flow improvements. The density improvement, expressed as a reduction in traffic density, reaches its largest magnitude around 30–40% sensing share. For smaller sensing regions, insufficient control action limits congestion mitigation. For larger sensing regions, the reduced participation in actuation weakens the controller’s ability to prevent density accumulation. The minimum density values thus coincide with the same intermediate sensing range that optimizes estimation and speed performance.

The share of vehicles actively participating in control increases strongly with sensing length up to approximately 35–40%, where participation peaks. This reflects an improved alignment between informed vehicles and the actuation region. For larger sensing shares, participation decreases significantly, indicating that the relative size of the actuation zone becomes insufficient to fully exploit the available information. This decline in active control contribution explains the concurrent reduction in traffic performance improvements for large sensing regions.

Across all performance metrics, the results consistently indicate the existence of an optimal sensing share of approximately 25–55% of the controlled section for this specific (bifurcation) case study. This optimum reflects a fundamental trade-off between observability and controllability. Short sensing sections lead to insufficient state awareness, whereas excessively long sensing regions reduce effective control authority within the actuation zone. An intermediate sensing configuration balances timely and relevant information with sufficient actuation coverage, thereby maximizing estimation accuracy and macroscopic traffic improvements. To conclude, the parametrization of V2VSL needs to be adjusted given the specific bottleneck and context. Yet, the parameters listed in Table 1 serve as a robust, good-enough starting point.

### Controller Performance Benchmark

In this section, we aim to contextualize the proposed V2VSL approach in a benchmark with alternative controllers, including analytic controllers, learning-based approaches with access to infrastructure-sensor information, and a decentralized, learning-based controller for CVs. We evaluate and compare the performance of all controllers at the three case studies, and for a multitude of performance measures.

#### Benchmark Controllers

The benchmark is designed to disentangle the effect of (i) control methodology and (ii) information structure. In particular, V2VSL is compared against centralized infrastructure-based controllers to assess whether decentralized V2V coordination can achieve competitive or superior performance without relying on fixed sensing infrastructure. The selected benchmark controllers are summarized in Table 2 and include:

- Analytic controllers.
  - Simple proportional speed controller (SPSC) Kušić et al. (2016).
  - Mainline virtual metering (MVM) Kušić et al. (2016).
  - Model predictive controller (MPC) Han et al. (2017).
- Learning-based controllers.
  - Learning-based, deep deterministic policy gradient (DDPG) Gregurić et al. (2022).
  - Bidirectional long short-term memory networks (BiLSTM) Song et al. (2025).
  - Duelling double deep Q-network (D3QN) Song et al. (2025).

The analytic controllers represent established baseline approaches in freeway traffic control, ranging from simple proportional feedback (SPSC), to flow-regulation inspired strategies (MVM), and optimization-based model predictive control (MPC). Together, they span increasing levels of modelling sophistication and computational complexity. The learning-based controllers represent contemporary deep reinforcement learning approaches for VSL control that leverage centralized infrastructure sensing and rich state representations. These methods serve as data-driven, performance-oriented benchmarks under V2I assumptions.

**Table 2:** Comparison VSL control algorithms for benchmark

Controller	Sensing	Communication	Architecture
SPSC (Kušić et al. 2016)	Loop detectors	–	Centralized
MVM (Kušić et al. 2016)	Loop detectors	–	Centralized
MPC (Han et al. 2017)	Loop detectors	–	Centralized
DDPG (Gregurić et al. 2022)	Loop detectors	–	Centralized
BiLSTM (Song et al. 2025)	CAVs	V2I	Centralized
D3QN (Song et al. 2025)	CAVs	V2I	Centralized
V2VSL	CAVs	V2V	Decentralized

The selected benchmark controllers were chosen to cover (i) classical feedback control, (ii) model-based optimal control, and (iii) modern deep reinforcement learning approaches under centralized V2I assumptions. This allows a systematic comparison across increasing levels of model sophistication and data-dependence. Importantly, while all benchmark learning-based methods rely on infrastructure-supported sensing or centralized information aggregation, the proposed V2VSL operates under a fully decentralized V2V communication paradigm. The benchmark therefore evaluates whether decentralized coordination can match or outperform centralized control architectures

The MVM controller emulates classical ramp metering on the freeway mainline by regulating the upstream inflow toward a bottleneck. Let $\rho _d(t)$ denote the measured downstream density (veh/km/lane) at time *t*, $\rho _{\textrm{cr}}$ the critical density corresponding to capacity, $q_{\textrm{out}}(t)$ the desired mainline flow (veh/h), and *C* the nominal bottleneck capacity. It computes a target flow according to

8 $$\begin{aligned} q_{\textrm{out}}(t) = C - K_I \big (\rho _d(t) - \rho _{\textrm{cr}}\big ) \end{aligned}$$

where $K_I>0$ is an integral (or regulation) gain.

The commanded speed limit $v_{\textrm{VSL}}(t)$ (km/h) is then derived from the fundamental relation q=ρv using the measured upstream density $\rho _u(t)$, i.e. $v_{\textrm{VSL}}(t) = q_{\textrm{out}}(t) / \rho _u(t)$.

The SPSC controller directly adjusts the speed limit proportionally to the density error. Let ρ(t) be the controlled section density and $\rho ^*$ the desired setpoint (typically close to $\rho _{\textrm{cr}}$). The SPSC law is given by

$$\begin{aligned} v_{\textrm{VSL}}(t) = v_{\max } - K_P\big (\rho (t) - \rho ^*\big ), \end{aligned}$$

where $v_{\max }$ is the free-flow speed limit and $K_P>0$ is a proportional gain. While MVM regulates flow indirectly through a metering analogy, SPSC applies direct proportional feedback on density to stabilize traffic near critical conditions.

The MPC controller uses an extended cell-transmission model (CTM) (Hegyi et al. 2005) that maintains linear properties while accurately capturing jam wave propagation. The optimal controller is derived by minimizing total travel time within the context of the CTM model, and formulated as a linear-quadratic optimization problem.

The DDPG controller uses a DDPG reinforcement learning framework with an actor-critic architecture to learn continuous speed limit actions and spatial configurations of dynamic speed limit zones from a rich image-like state representation of connected vehicle traffic; the actor network directly outputs a continuous action vector (e.g., differential speed limits and spatial parameters of zones) based on current traffic state, while the critic evaluates these actions using a reward designed to promote mainstream speed homogenization and higher throughput at congested bottlenecks, allowing the controller to adjust both speed limits and zone positions to improve motorway flow compared to baseline control methods.

The BiLSTM controller uses BiLSTM networks to process connected vehicle data and learn spatiotemporal traffic patterns: it takes time-series of traffic states (e.g., densities and speeds across lanes collected by CVs) and produces future state predictions that capture both forward and backward temporal dependencies, enabling the VSL controller to anticipate how traffic conditions will evolve rather than react only to the current snapshot.

The D3QN controller leverages a reinforcement learning agent framework, where the D3QN approximates the action-value function *Q*(*s*, *a*) using two streams (value and advantage) to better estimate the expected utility of lane-specific speed limit actions *a* in state *s*, and incorporates double Q-learning to reduce overestimation bias during training; this agent learns a policy that outputs differentiated speed limits per lane to optimize a cumulative reward reflecting traffic efficiency and flow homogenization. BiLSTM supplies predictive spatio-temporal state features that make the decision process forward-looking, while D3QN uses those features to learn a robust lane-specific VSL policy.

*Implementation details:* All analytic controllers and the infrastructure-based learning controller DDPG have access to speed sensors at regular intervals (2 min). The speed sensors (loop detectors) are placed on all sensors an the beginning of the sensing section. The connected-vehicle based controllers BiLSTM and D3QN are assumed to enable communication between vehicles every two seconds (to be comparable with V2VSL, and to exchange information including vehicle speed, and position information. Contrary to BiLSTM and D3QN that rely on a V2I-based communication system, V2VSL fully works with a V2V-based communication system. Parameters and implementation taken from the respective works.

#### Benchmark Results

The benchmark results across all three case studies (bifurcation, on-ramp merge, and lane-drop) are reported in Table 3, and reveal three consistent patterns.

First, all control strategies significantly outperform the no-control scenario across all performance metrics. Speed and flow increase consistently, while density and total travel time (TTT) decrease. The magnitude of improvement becomes particularly pronounced in the more severe bottleneck scenarios (case studies 2 and especially 3), indicating that VSL control is most beneficial under congested conditions. Among all controllers, the learning-based methods—particularly D3QN and BiLSTM—achieve the largest absolute improvements. For instance, D3QN reduces TTT by up to − 11.46%, − 8.87%, and − 14.57%, respectively. This confirms that deep reinforcement learning, when supported by centralized infrastructure sensing and rich state information, can exploit complex spatio-temporal traffic dynamics to extract maximal performance gains.

Second, as expected, learning-based controllers slightly outperform the analytical controllers across all case studies. The performance hierarchy is consistent: D3QN ≥ BiLSTM ≥ DDPG ≥ MPC ≥ MVM ≥ SPSC. MPC remains the strongest analytical approach and performs competitively, often approaching the learning-based controllers. However, the learning-based methods achieve additional gains of several percentage points in TTT reduction and throughput improvement. This performance advantage, however, comes at a cost, such as high-dimensional state representations, centralized data aggregation, infrastructure-supported sensing (loop detectors or V2I communication), and significant computational and training efforts. Thus, while learning-based approaches provide the highest peak performance, they require substantial infrastructure and computational complexity, that might not justify the minor improvements over analytic approaches.

Third, the key result of this benchmark is that V2VSL performs remarkably well despite operating under a fundamentally different information and architecture paradigm. Unlike all other controllers, V2VSL requires no fixed sensing infrastructure, does not rely on V2I communication, is fully decentralized, is computationally lightweight, and does not require model calibration or expensive learning-based training. Yet, it achieves: up to − 8.57%, − 5.83%, respectively − 9.85% TTT reduction (given 100% compliance), which places it consistently close to the more complex analytical controller MVM and, in some cases, near MPC. This is particularly notable because V2VSL does not optimize a global objective via centralized optimization or reinforcement learning. Instead, it relies on decentralized vehicle-to-vehicle coordination and simple local interaction rules. Despite this simplicity, the emergent system-level behaviour yields substantial congestion mitigation. In other words, V2VSL achieves a large fraction of the achievable performance improvement at only a fraction of the complexity and infrastructure cost.

**Table 3:** VSL controller benchmark. The controllers V2VSL, SPSC, MPS, DDPG, BiLSTM, and D3QN are compared against the no control case for three case studies CS1 (bifurcation), CS2 (on-ramp merge), and CS3 (lane-drop). Values for speed (km/h), flow (veh/h), density (veh/km), and total travel time (TTT, veh x h) reported as average with standard deviation in brackets as result of 20 SUMO microsimulations with different random seeds. Values below brackets represent relative change to no controll scenario

		No controll	SPSC (Anal.)	MVM (Anal.)	MPC (Anal.)	DDPG (Learn.)	BiLSTM (Learn.)	D3QN (Learn.)	V2VSL (50%)	V2VSL (100%)
*Case study 1*	Speed	68.21 [3.23]	70.10 [4.41] +2.77%	73.20 [4.75] +7.32%	74.25 [5.10] +8.86%	74.65 [5.62] +9.44%	74.83 [5.81] +9.70%	74.91 [5.95] +9.82%	70.32 [4.56] +3.09%	73.16 [4.84] +7.26%
	Flow	2597.04 [213.48]	2668.73 [248.61] +2.76%	2770.23 [284.22] +6.67%	2805.55 [295.02] +8.03%	2825.76 [310.53] +8.81%	2838.99 [322.46] +9.32%	2845.33 [330.11] +9.57%	2674.95 [253.74] +3.00%	2778.83 [285.94] +7.00%
	Density	167.26 [12.52]	163.02 [14.83] −2.54%	157.25 [17.25] −5.99%	155.13 [18.01] −7.24%	154.55 [19.21] −7.60%	154.12 [19.02] −7.85%	153.89 [20.02] −8.00%	162.74 [14.92] −2.70%	156.93 [17.45] −6.18%
	TTT	96.07 [5.34]	90.25 [6.20] −6.06%	87.91 [6.86] −8.49%	86.75 [7.02] −9.70%	86.24 [7.52] −10.23%	86.01 [7.70] −10.47%	85.06 [7.94] −11.46%	90.03 [6.53] −6.29%	87.84 [6.84] −8.57%
*Case Study 2*	Speed	64.95 [3.56]	66.10 [3.98] +1.77%	68.35 [4.22] +5.23%	69.25 [4.48] +6.62%	69.85 [4.76] +7.54%	70.05 [4.91] +7.85%	70.18 [5.02] +8.05%	66.25 [4.05] +2.00%	68.20 [4.30] +5.00%
	Flow	1449.50 [175.24]	1505.80 [192.40] +3.88%	1578.20 [205.33] +8.19%	1605.75 [214.86] +10.09%	1620.40 [226.95] +11.10%	1632.10 [233.44] +12.60%	1639.85 [241.18] +13.13%	1512.30 [198.75] +4.33%	1585.90 [209.52] +9.41%
	Density	91.52 [3.24]	89.90 [3.60] −1.77%	87.45 [3.82] −4.45%	86.60 [4.05] −5.38%	86.05 [4.22] −5.98%	85.80 [4.34] −6.25%	85.65 [4.48] −6.41%	89.75 [3.72] −1.93%	87.10 [3.95] −4.83%
	TTT	55.91 [3.52]	53.95 [3.88] −3.51%	52.70 [4.05] −5.74%	52.10 [4.18] −6.82%	51.75 [4.32] −7.44%	51.60 [4.41] −7.71%	50.95 [4.56] −8.87%	54.05 [3.95] −3.33%	52.65 [4.12] −5.83%
*Case Study 3*	Speed	68.77 [3.41]	70.95 [4.60] +3.17%	72.85 [4.88] +5.93%	73.90 [5.15] +7.46%	74.35 [5.52] +8.11%	74.62 [5.74] +8.51%	74.88 [5.93] +8.88%	71.10 [4.67] +3.39%	72.95 [4.92] +6.08%
	Flow	3912.62 [285.71]	4015.30 [310.42] +2.63%	4148.75 [338.60] +6.04%	4205.40 [351.22] +7.48%	4248.65 [365.80] +8.59%	4282.91 [378.44] +9.47%	4310.55 [389.77] +10.17%	4040.15 [318.20] +3.26%	4160.80 [342.55] +6.35%
	Density	240.50 [19.83]	232.40 [21.55] −3.37%	225.85 [23.44] −6.09%	223.10 [24.12] −7.23%	221.75 [25.30] −7.80%	220.60 [26.02] −8.27%	219.20 [27.11] −8.86%	231.05 [22.04] −3.93%	226.90 [23.70] −5.66%
	TTT	145.31 [11.78]	135.60 [12.95] −6.68%	130.40 [13.80] −10.26%	128.25 [14.25] −11.74%	126.90 [14.92] −12.67%	125.80 [15.30] −13.43%	124.15 [15.88] −14.57%	136.10 [13.10] −6.34%	131.00 [13.75] −9.85%

The comparison between 50% and 100% compliance clearly demonstrates the importance of penetration rate. Across all case studies, 100% compliance consistently outperforms 50% compliance. The performance gap is moderate but systematic (typically 2–4 percentage points in TTT). Even at 50% compliance, V2VSL still provides meaningful improvements over no control and often outperforms SPSC. This indicates that the method is scalable and robust to partial penetration. Benefits grow smoothly with adoption. Full compliance is not strictly necessary for effectiveness.

The benchmark demonstrates a clear trade-off between maximum achievable performance (learning-based centralized controllers), and architectural simplicity, deployability, and infrastructure independence (V2VSL). The results suggest that decentralized V2V coordination is not merely a low-cost fallback solution, but a feasible, viable, and scalable alternative to infrastructure-heavy V2I-based control architectures, achieving substantial congestion mitigation with dramatically reduced system complexity.

## Conclusions

This work introduced V2VSL, a fully decentralized, infrastructure-free approach to VSL. It relies on connected vehicles that serve as a decentralized communication, sensing, and actuation infrastructure. Compliant vehicles/drivers that follow the suggestions decelerate slightly to mitigate congestion formation downstream and reach their destinations faster. Unlike existing infrastructure-based and model-dependent VSL systems, the proposed method requires no roadside equipment or global traffic state estimation, but instead relies on lightweight consensus and gossip algorithms for decentralized speed estimation combined with a Bellman bang-bang control law.

The results of simulation experiments demonstrate that the method succeeds in mitigating congestion, achieving up to 15% higher speed, 5% lower density, and 8% higher outflow compared to the uncontrolled scenario. The achieved improvements are of similar order of magnitude to those reported in prior infrastructure-based VSL studies, despite the absence of roadside sensing, centralized coordination, or explicit traffic models. This highlights the potential of decentralized, infrastructure-free V2V coordination as a viable alternative rather than a direct replacement for centralized VSL systems.

Rather than competing with model-predictive or learning-based VSL approaches on optimality, this work demonstrates that a simple, decentralized, and infrastructure-free control concept can be sufficient to mitigate congestion in realistic bottleneck scenarios. The results suggest that feasibility, robustness, and deployability constitute an important and often under-explored dimension in traffic control research, complementing performance-driven approaches.

The proposed VSL approach acts robust to inaccurate, outdated estimates of the mean speed in the ROI, and reliably recovers from disruptions due to gaps between platoons. Importantly, improvements become significant at a compliance rate as low as 25%, making the method potentially viable in near-term mixed traffic environments with partial CV penetration.

The findings suggest that decentralized V2V coordination can deliver performance comparable to centralized, infrastructure-heavy systems, while being more robust to disconnection and less costly to deploy. Nevertheless, this work’s findings and chosen parameters are limited to the simulation case study.

### Limitations

The approach itself is limited by distances and the connected vehicles’ communication technology:

- If the communication distance is too short, the decentralized communication infrastructure lacks sufficient connectivity for the consensus and gossip algorithms;
- if the actuation section is too long, estimation quality suffers from aged information;
- if the sensing section too short, estimation quality suffers as the time that vehicles participate in the consensus algorithm is too short.

### Avenues for Future Research

Future research could delve into …

- Assessing decentralized speed estimation on a lane level (differential VSL),
- Investigate the robustness of parameter optimization in different real-world contexts (i.e. varying bottlenecks, distances, communication technologies),
- Elaborating on methods for parameter estimation in real-world contexts,
- Studying the effect of platoons, longer actuation areas, and shorter communication distances $d_c$ on the control performance,
- And benchmark the performance against various learning-based approaches.
- Moreover, the design of effective incentive mechanisms to encourage more drivers to comply (i.e. rewards, gamification, information on decongestion benefits) might be an interesting question to study.

## Data Availability

An open-source implementation and computational results are provided as SUMO simulation with Python on GitHub: https://github.com/DerKevinRiehl/decentralized_vsl/.

## Footnotes

1 https://inrix.com/scorecard/.
